# IKK***β***-Targeted Anti-Inflammatory Activities of a Butanol Fraction of Artificially Cultivated *Cordyceps pruinosa* Fruit Bodies

**DOI:** 10.1155/2014/562467

**Published:** 2014-07-15

**Authors:** Han Gyung Kim, Woo Seok Yang, Gi-Ho Sung, Ji Hye Kim, Gwang-Soo Baek, Eunji Kim, Sungjae Yang, Yung Chul Park, Jae Mo Sung, Deok Hyo Yoon, Tae Woong Kim, Sungyoul Hong, Jong-Hoon Kim, Jae Youl Cho

**Affiliations:** ^1^Department of Genetic Engineering, Sungkyunkwan University, Suwon 440-746, Republic of Korea; ^2^Mushroom Research Division, National Institute of Horticultural and Herbal Science, Rural Development Administration, Eumseong 369-873, Republic of Korea; ^3^Department of Forest Environment Protection, Kangwon National University, Chuncheon 200-701, Republic of Korea; ^4^Institute of Mushroom, Mushtech, Chuncheon 200-180, Republic of Korea; ^5^Department of Biochemistry, Kangwon National University, Chuncheon 200-701, Republic of Korea; ^6^Department of Veterinary Physiology, College of Veterinary Medicine, Biosafety Research Institute, Chonbuk National University, Jeonju 561-756, Republic of Korea

## Abstract

The inhibitory activities of the *Cordyceps pruinosa* butanol fraction (Cp-BF) were investigated by determining inflammatory responses of lipopolysaccharide (LPS)-treated RAW264.7 macrophage cells and by evaluating HCl/ethanol (EtOH)-triggered gastric ulcers in mice. The molecular mechanisms of the inhibitory effects of Cp-BF were investigated by identifying target enzymes using biochemical and molecular biological approaches. Cp-BF strongly inhibited the production of NO and TNF-*α*, release of reactive oxygen species (ROS), phagocytic uptake of FITC-dextran, and mRNA expression levels of interleukin (IL)-6, inducible NO synthase (iNOS), and tumour necrosis factor-alpha (TNF)-*α* in activated RAW264.7 cells. Cp-BF also strongly downregulated the NF-*κ*B pathway by suppressing IKK*β* according to luciferase reporter assays and immunoblot analysis. Furthermore, Cp-BF blocked both increased levels of NF-*κ*B-mediated luciferase activities and phosphorylation of p65/p50 observed by IKK*β* overexpression. Finally, orally administered Cp-BF was found to attenuate gastric ulcer and block the phosphorylation of I*κ*B*α* induced by HCl/EtOH. Therefore, these results suggest that the anti-inflammatory activity of Cp-BF may be mediated by suppression of IKK*α* and its downstream NF-*κ*B activation. Since our group has established the mass cultivation conditions by developing culture conditions for *Cordyceps pruinosa*, the information presented in this study may be useful for developing new anti-inflammatory agents.

## 1. Introduction

Inflammation is a complicated defensive response against various infecting pathogens and toxins and serves as one of the body's innate immunity barriers. As a major inflammatory cell population, macrophages respond to inflammatory events by phagocytosing infected materials and producing cytokines such as interleukin (IL)-1 and tumour necrosis factor (TNF)-*α*, as well as inflammatory mediators such as nitric oxide (NO) and prostaglandin E_2_ (PGE_2_). For these responses, macrophages need to activate cell surface receptors (e.g., toll-like receptors) by interacting with their ligands, such as lipopolysaccharide (LPS) from Gram (−) bacteria. Extracellular signals received by plasma membrane receptors will then generate intracellular signalling events through nonreceptor type protein tyrosine kinases, including Syk, Src, phosphoinositide-3-kinase (PI3K), and mitogen activated protein kinase (MAPK), upstream of extracellular signal-related kinase (ERK), p38, and c-Jun N-terminal kinase (JNK). Ultimately, all of these intracellular responses reach activation of transcription factors including CREB, nuclear factor-*κ*B (NF-*κ*B), and activator protein-1 (AP-1) [1, 2]. The outcomes of such activation are linked to the expression of various proinflammatory genes and inflammatory mediators [3–5]. In addition, recent findings have raised the possibility that sustained inflammatory responses can cause serious diseases such as septic shock, cancer, diabetes, gastritis, and atherosclerosis[5–7]. Therefore, development of stronger and safer anti-inflammatory remedies may contribute to more promising strategies to effectively treat inflammation-derived diseases.

The genus* Cordyceps*, which includes* Cordyceps sinensis*,* Cordyceps militaris*,* Cordyceps pruinosa*, and* Cordyceps bassiana*, comprises ethnopharmacologically valuable mushrooms in Korea, China, and Japan [8]. The genus* Cordyceps* has been traditionally used for the treatment of various inflammatory and infectious diseases, such as eczema, skin diseases, chronic bronchitis, asthma, and tuberculosis, or used as a tonic for longevity, endurance, and vitality [8, 9]. The mushroom* Cordyceps* was also described in ancient traditional Chinese medicine literatures as a medicinal ingredient prescribed for relieving chronic bronchitis, chronic obstructive pulmonary disease, and tuberculosis [10, 11]. Additional studies have found that these mushrooms have numerous biological activities based on antioxidative, anticancer, antidiabetes, antibacterial, antifungal, and antifatigue properties [12, 13]. From phytochemical approaches, it has been reported that cordycepin, ergosterol peroxide, and polysaccharides are the major components responsible for the various pharmacological activities of* Cordyceps* [14]. However, in spite of their excellent pharmaceutical and nutraceutical potential, it is difficult to mass produce these mushrooms. To overcome the limitations of mass production of* Cordyceps* species, our group has developed an artificial culturing condition with a grain rice-enriched medium that is able to achieve mass production of fruit bodies of* Cordyceps militaris*,* Cordyceps pruinosa*, and* Cordyceps bassiana* under defined culture conditions.

**Figure 1:**
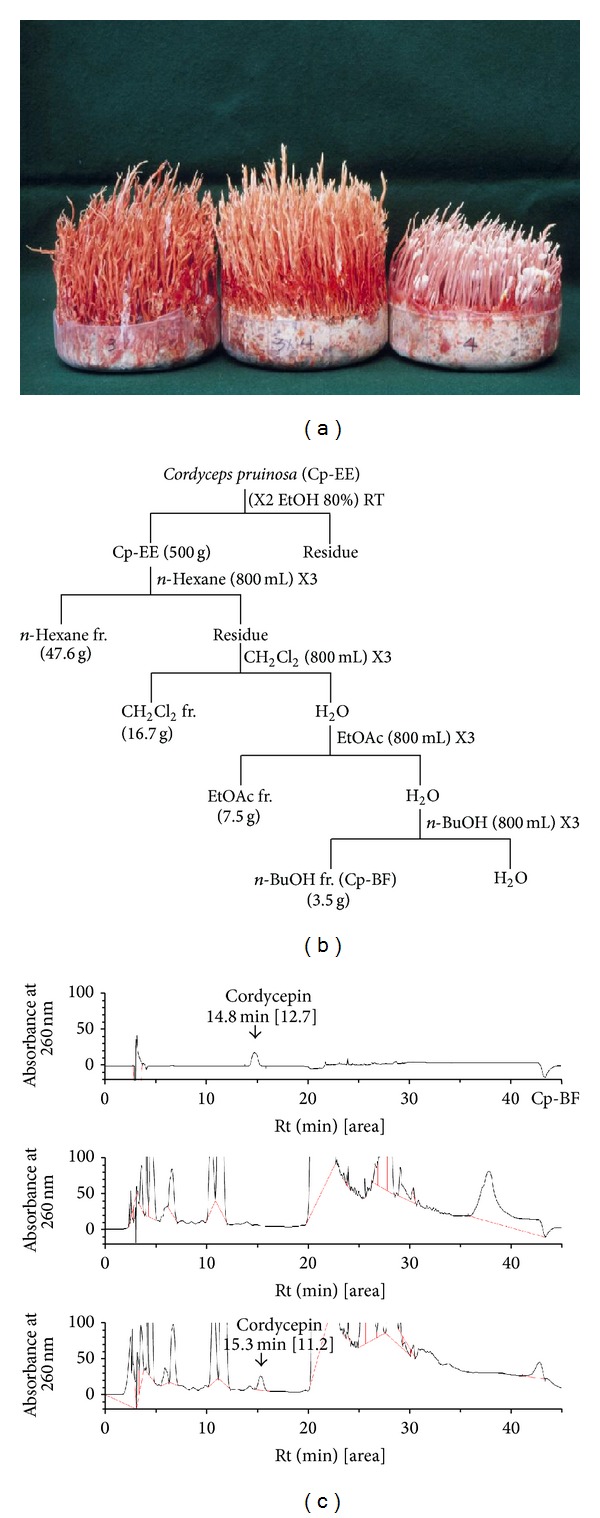
Description and HPLC-analysis of the butanol fraction of cultivated* Cordyceps pruinosa*. (a) Photos of the artificially cultivated form of* Cordyceps pruinosa*. (b) Experimental procedure for preparation of the butanol fraction from* Cordyceps pruinosa* (Cp-BF). (c) Cp-BF and cordycepin were analysed by high performance liquid chromatography (HPLC) equipped with KNAUER. The elution solvents were distilled water and acetonitrile. The gradient step of the solvent was “water to acetonitrile 1%/min” performed using a Vydac C18 Column.

Among the mushrooms that we demonstrated to have grown in a mass production setting, we focused especially on* Cordyceps pruinosa* (Figure 1(a)), as only a few papers have reported its pharmacological activities towards cancer and inflammation, [15, 16] and the cultivation rate of its fruit body is much higher than other species. Although the anti-inflammatory activity of the methanol extract of* Cordyceps pruinosa* has been suggested previously by determination of IL-1*β*, TNF-*α*, NO, and PGE_2_ levels [15], the exact molecular mechanism for these effects has not been fully elucidated. Therefore, in this study,we aimed to explore anti-inflammatory mechanism of* Cordyceps pruinosa* butanol fraction by using kinase assays, luciferase reporter assays, molecular binding assays, and overexpression strategies, as well as a mouse model of gastritis.

## 2. Materials and Methods

### 2.1. Materials

Artificially cultivated fresh fruiting bodies of* Cordyceps pruinosa* were obtained from Mushtech Co. (Chuncheon, Korea) and were authenticated by Dr. J. M. Sung (Mushtech, Chuncheon, Korea). A voucher specimen (EFCC #11968) was deposited in the Entomopathogenic Fungal Culture Collection, Kangwon National University, Korea. Forskolin, cordycepin, (3-4,5-dimethylthiazol-2-yl)-2,5-diphenyltetrazolium bromide (MTT), H_2_DCFDA, fluorescein isothiocyanate (FITC)-dextran, phorbol 12-myristate 13-acetate (PMA), and LPS from* Escherichia coli* 0111:B4 were purchased from Sigma-Aldrich (St. Louis, MO). BAY 11-7082 was obtained from Calbiochem (La Jolla, CA). Luciferase constructs containing binding promoters for NF-*κ*B, CREB, and AP-1 were gifts from Professor Chung, Hae Young (Pusan National University, Pusan, Korea) and Man Hee Rhee (Kyungpook National University, Daegu, Korea). Enzyme immunosorbent assay (ELISA) kits for determining levels of TNF-*α* were purchased from Amersham (Little Chalfont, Buckinghamshire, UK). Foetal bovine serum (FBS) and RPMI1640 were obtained from GIBCO (Grand Island, NY). RAW264.7 and HEK293 cells were purchased from ATCC (Rockville, MD). All other chemicals were of Sigma-Aldrich grade. Phosphospecific and total antibodies for transcription factors (p65, p50, and c-Jun), MAPK (ERK, p38, and JNK), I*κ*B*α*, IKK*β*, AKT, lamin A/C, and *β*-actin were obtained from Cell Signalling Technology (Beverly, MA). Primers (Table 1) were designed in our laboratory and were synthesized by Bioneer (Daejeon, Korea).

### 2.2. Preparation and Characteristics of Cp-BF

After drying the fruiting bodies of* Cordyceps pruinosa* at 50°C, an ethanol extract was prepared and several solvent fractions were subsequently prepared with* n*-hexane,* n*-butanol, and ethyl acetate. After evaporation of the fractions under reduced pressure, each fraction was dried using a freeze-dryer to give solid subfractions. Among the various fractions, the butanol fraction (Cp-BF) gave a 7% yield. The phytochemical characteristics of Cp-BF were identified by high performance liquid chromatography (HPLC) analysis as reported previously [17]; the system was equipped with KNAUER (Wellchrom HPLC-pump, K-1001, Wellchrom fast scanning spectrophotometer K-2600, and 4 channel degasser K-500). The elution solvents consisted of distilled water and acetonitrile. The gradient step for solvent elution was “water to acetonitrile 1%/min” using a Vydac C18 column.

### 2.3. Mice

Six-week-old male ICR mice (6–8 weeks old, 17–21 g) were obtained from Daehan Biolink (Osong, Korea) and maintained in plastic cages under conventional conditions. Water and pelleted diets (Samyang, Daejeon, Korea) were supplied* ad libitum*. Studies were performed in accordance with guidelines established by the Sungkyunkwan University Institutional Animal Care and Use Committee.

### 2.4. Cell Culture

RAW264.7 and HEK293 cells were cultured with RPMI1640 medium supplemented with 10% heat-inactivated FBS, glutamine, and antibiotics (penicillin and streptomycin) at 37°C in a 5% CO_2_ atmosphere. For each experiment, cells were detached with a scraper. Examination of cell densities at 2 × 10^6^ cells/mL revealed that the proportion of dead cells was consistently <1% according to Trypan blue dye exclusion as the criterion for viability.

### 2.5. NO and TNF-*α* Production

After preincubation of RAW264.7 cells (1 × 10^6^ cells/mL) for 18 h, cells were pretreated with Cp-BF (0–200 *μ*g/mL) for 30 min and then incubated with LPS (1 *μ*g/mL) for an additional 24 h. The inhibitory effect of Cp-BF on NO and TNF-*α* production was determined by analysing NO and TNF-*α* levels with Griess reagent and ELISA kits as described previously [18, 19].

### 2.6. Determination of Phagocytic Uptake

Phagocytic activity of RAW264.7 cells was determined as described previously, with some modifications [20]. Briefly, RAW264.7 (5 × 10^4^) cells treated with Cp-BF (0–200 *μ*g/mL) were resuspended in 100 *μ*L PBS containing 1% human AB serum and incubated with fluorescein isothiocyanate (FITC)-dextran (1 mg/mL) at 37°C for 2 h. Incubations were stopped by adding 2 mL ice-cold phosphate-buffered saline (PBS) containing 1% human serum and 0.02% sodium azide. The cells were then washed three times with cold PBS-azide and analysed on a FACScan flow cytometer as reported previously [21].

### 2.7. Determination of Reactive Oxygen Species Generation

Levels of intracellular ROS were determined by analysing the change in fluorescence resulting from the oxidation of the fluorescent probe H_2_DCFDA. Briefly, 5 × 10^5^ RAW264.7 cells were exposed to Cp-BF (0–100 *μ*g/mL) for 30 min. After incubation, cells were incubated with LPS (1 *μ*g/mL) as an inducer of ROS production at 37°C for 6 h. Cells were then incubated with 50 *μ*M H_2_DCFDA for 1 h at 37°C. The degree of fluorescence, corresponding to the level of intracellular ROS, was determined using a FACScan flow cytometer (Beckton-Dikinson, San Jose, CA, USA) as reported previously [16].

### 2.8. Flow Cytometric Analysis

RAW264.7 cells (2 × 10^6^ cells/mL) treated with Cp-BF (0–100 *μ*g/mL) and FITC-dextran or H_2_DCFDA were washed with a staining buffer (containing 2% rabbit serum and 1% sodium azide in PBS) and incubated with directly labelled antibodies for a further 45 min on ice. After washing three times with staining buffer, cells were analysed on a FACScan flow cytometer (Becton-Dickinson).

### 2.9. Cell Viability Test

After preincubation of RAW264.7 cells (1 × 10^6^ cells/mL) for 18 h, the Cp-BF (0–200 *μ*g/mL) was added to the cells and incubated for an additional 24 h. The cytotoxic effect of the Cp-BF was then evaluated by a conventional MTT assay as described previously [22]. Briefly, 3 h prior to culture termination, 10 *μ*L of a MTT solution (10 mg/mL in phosphate buffered saline, pH 7.4; PBS) was added and cells were continuously cultured until termination of the experiment. Incubation was halted by the addition of 15% sodium dodecyl sulphate into each well to solubilise the produced formazan [23]. The absorbance at 570 nm (OD_570_) was measured using a Spectramax 250 microplate reader.

### 2.10. mRNA analysis by Reverse Transcription (RT) Polymerase Chain Reaction (PCR)

To determine cytokine mRNA expression levels, total RNA was isolated from LPS-treated RAW264.7 cells with TRIzol Reagent (Gibco BRL) according to the manufacturer's instructions. Total RNA was stored at −70°C until use. Determination of mRNA levels was performed using RT-PCR as reported previously [24]. Results are expressed as the ratio of optimal density to glyceraldehyde 3-phosphate dehydrogenase (GAPDH). The sequences of the primers used in this analysis are shown in Table 1.

### 2.11. Transfection of DNA and Luciferase Reporter Gene Activity Assay

HEK293 or RAW264.7 cells (5 × 10^6^ cells/mL) were transfected with 1 *μ*g/mL of empty vectors or FLAG-IKK*β*. Transfections were performed with lipofectamine 2000 (Invitrogen, Grand Island, NY) in 100 mm cell culture dishes in the presence or absence of MHNC. For luciferase assays, HEK293 cells (1 × 10^6^ cells/mL) were transfected with 1 *μ*g of plasmids containing NF-*κ*B-Luc, CREB-Luc, or AP-1-Luc, as well as *β*-galactosidase using the PEI method [25, 26] in 12-well plates according to the manufacturer's protocol. Cells were used for experiments 48 h after transfection. Luciferase assays were performed using the Luciferase Assay System (Promega, Madison, WI) as reported previously [27].

### 2.12. Preparation of Total Lysates and Nuclear Fractions, Immunoblotting, and Immunoprecipitation

Stomach tissues or cultured cells (HEK293 and RAW264.7) (5 × 10^6^ cells/mL) were washed three times in cold PBS with 1 mM sodium orthovanadate and lysed in lysis buffer (20 mM Tris-HCl, pH 7.4, 2 mM EDTA, 2 mM ethylenediaminetetraacetic acid, 50 mM *β*-glycerophosphate, 1 mM sodium orthovanadate, 1 mM dithiothreitol, 1% Triton X-100, 10% glycerol, 10 *μ*g/mL aprotinin, 10 *μ*g/mL pepstatin, 1 mM benzamide, and 2 mM phenylmethanesulfonyl fluoride) for 30 min with rotation at 4°C. Next, lysates were clarified by centrifugation at 16,000 ×g for 10 min at 4°C and stored at −20°C until needed.

Nuclear lysates were prepared in a three-step procedure [28]. After treatment, cells were collected with a rubber policeman, washed with 1 × PBS, and lysed in 500 *μ*L of lysis buffer on ice for 4 min. Cell lysates were then centrifuged at 19,326 ×g for 1 min in a microcentrifuge. In the second step, the pellet (the nuclear fraction) was washed once in washing buffer, which was the same as the lysis buffer with the omission of Nonidet P-40. In the final step, nuclei were treated with an extraction buffer containing 500 mM KCl, 10% glycerol, and the inhibitors noted in the lysis buffer. The nuclei/extraction buffer mixture was frozen at −80°C and then thawed on ice and centrifuged at 19,326 ×g for 5 min. Finally, the supernatant was collected as the nuclear extract.

For immunoprecipitation, cell lysates containing equal amounts of protein (500 *μ*g) from RAW264.7 cells (1 × 10^7^ cells/mL) treated with or without LPS (1 *μ*g/mL) for 2.5 min were pre-cleared with 10 *μ*L of protein A-coupled Sepharose beads (50% v/v) (Amersham, Buckinghamshire, UK) for 1 h at 4°C. Precleared samples were then incubated with 5 *μ*L of anti-IKK*β* antibody overnight at 4°C. Immune complexes were mixed with 10 *μ*L of protein A-coupled Sepharose beads (50% v/v) and rotated for 3 h at 4°C.

Soluble cell lysates or boiled beads used for immunoprecipitation were subjected to immunoblotting for detection of phosphorylated or total levels of transcription factors (p65, p50, and c-Jun), MAPK (ERK, p38, and JNK), I*κ*B*α*, IKK*α*/*β*, IKK*β*, Akt, *γ*-tubulin, lamin A/C, and *β*-actin. Immunoblots were visualized as previously reported [29].

### 2.13. IKK*α* and IKK*β* Kinase Assays

To evaluate the ability of extracts to inhibit IKK*α* and IKK*β* kinase activities using purified enzymes, a kinase profiler service from Millipore (Billerica, MA) was used. In a final reaction volume of 25 *μ*L, IKK*α* or IKK*β* (human; 1–5 mU) was incubated with the reaction buffer and the reaction was initiated by the addition of MgATP. After incubation for 40 min at room temperature, the reaction was stopped by the addition of 5 mL of 3% phosphoric acid solution. Next, 10 *μ*L of the reaction product was spotted onto a P30 filtermat and washed three times for 5 min each in 75 mM phosphoric acid. The filtermats were washed once in methanol, dried, and then subjected to scintillation counting.

### 2.14. HCl/Ethanol (EtOH)-Induced Gastritis

Inflammation of the stomach was induced with HCl/EtOH, according to a published method [30]. Fasted ICR mice were orally treated with Cp-BF (200 mg/kg) or ranitidine (40 mg/kg) twice per day for 3 days. Thirty min after the final injection, 400 *μ*L of 60% EtOH in 150 mM HCl was administered orally. Each animal was anesthetized with an overdose of urethane 1 h after the administration of necrotizing agents. The stomach was then excised and gently rinsed under running tap water. After opening the stomach along the greater curvature and spreading it out on a board, the area (mm^2^) of mucosal erosive lesions was measured using a pixel-counter under blind condition as reported previously [31].

### 2.15. Statistical Analyses

Data are expressed as the mean ± standard deviation (SD) calculated from at least three independent experiments, each performed in triplicate, or representative of three different experiments with similar results. For statistical comparisons, results were analysed using analysis of variance/Scheffe's post hoc test and Kruskal-Wallis/Mann-Whitney test. Values of *P* < 0.05 were taken to indicate statistically significant differences. All statistical tests were carried out using the SPSS computer program (SPSS, Chicago, IL).

## 3. Results

### 3.1. Cp-BF Suppresses the Inflammatory Responses of Macrophages

**Figure 2:**
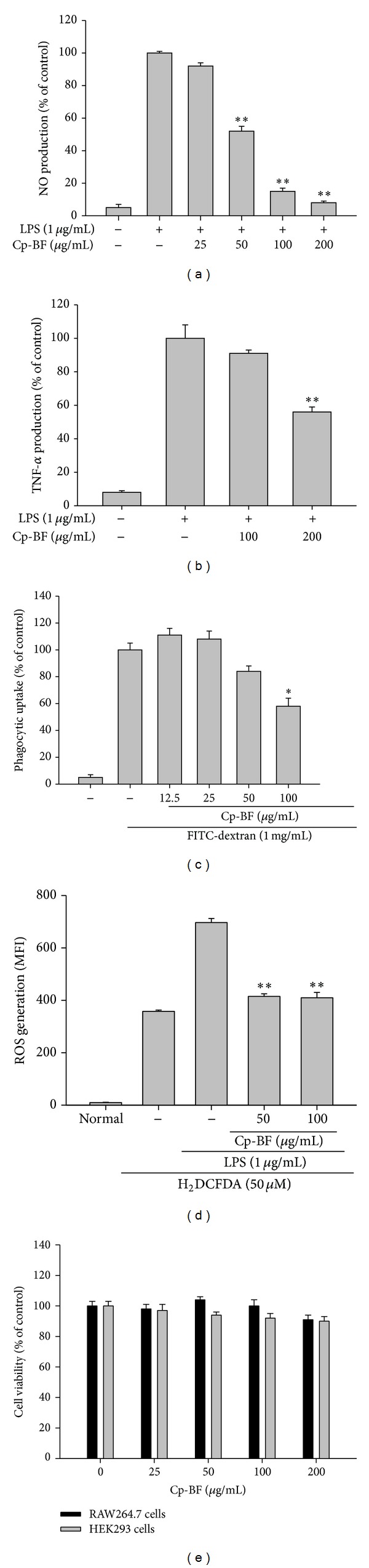
Effect of Cp-BF on production of inflammatory mediators, release of reactive oxygen species, and phagocytic uptake. ((a) and (b)) Levels of NO and TNF-*α* were determined by Griess assay and ELISA from culture supernatants of RAW264.7 cells treated with Cp-BF and LPS (1 *μ*g/mL) for 24 h. (c) The effect of Cp-BF on phagocytic uptake of RAW264.7 cells was determined by treatment with FITC-dextran (1 mg/mL) for 2 h and the uptake of which was determined by flow cytometric analysis. (d) The effect of Cp-BF on reactive oxygen species (ROS) generation in LPS-treated RAW264.7 cells was determined by incubation with H_2_DCFDA (50 *μ*M) and flow cytometric analysis. (e) Cell viability of RAW264.7 and HEK293 cells as determined by MTT assay. *P < 0.05 and **P < 0.01 compared to control.

We first tested the effect of Cp-BFin macrophages. As shown in Figures 2(a) and 2(b), Cp-BF dose-dependently suppressed the production of NO and TNF-*α* stimulated from LPS-treated RAW264.7 cells. In particular, 200 *μ*g/mL of Cp-ME greatly inhibited the production of NO up to 95% and the release of TNF-*α* by 43%. In addition, this fraction significantly decreased the uptake of FITC-dextran by up to 41% at 100 *μ*g/mL (Figure 2(c)). Moreover, Cp-BF almost completely suppressed the generation of ROS stimulated by LPS at 50 and 100 *μ*g/mL (Figure 2(d)). Importantly, Cp-BF at 200 *μ*g/mL did not affect the viability of either RAW264.7 or HEK293 cells (Figure 2(e)).

### 3.2. Cp-BF Suppresses the Inflammatory Responses at the Transcription Level

**Figure 3:**
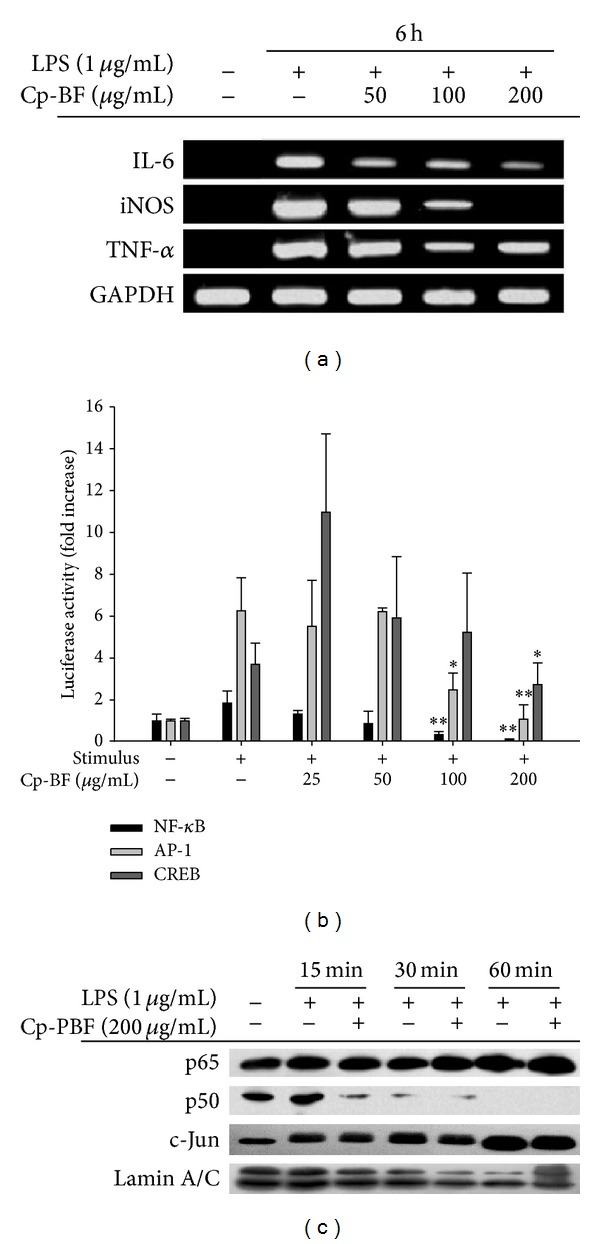
Effect of Cp-BF on the transcriptional regulation of inflammatory genes. (a) The mRNA levels of iNOS, IL-6, and TNF-*α* were determined by semiquantitative RT-PCR. (b) HEK293 cells cotransfected with plasmid constructs NF-*κ*B-Luc, CREB-Luc, or AP-1-Luc (each 1 *μ*g/mL), and *β*-gal (as a transfection control) were treated with Cp-BF in the presence or absence of PMA (100 nM; for NF-*κ*B and AP-1-Luc) or forskolin (100 nM for CREB-Luc). Luciferase activity was measured by a luminometer. (c) Levels of nuclear NF-*κ*B (p65 and p50) or AP-1/c-Jun were determined by immunoblot analysis of the nuclear fractions of LPS-treated RAW264.7 cells. *P < 0.05 and **P < 0.01 compared to control.

To determine the mechanism by which Cp-BF exerts its anti-inflammatory effects, we next analysed the mRNA levels of genes involved in inflammation. As shown in Figure 3(a) Cp-BF strongly suppressed the expression of iNOS and IL-6 between 100 and 200 *μ*g/mL, while this fraction weakly inhibited the mRNA expression of TNF-*α* at these concentrations. The regulatory role of Cp-BF on the activation of inflammatory transcription factors was determined by luciferase assay. Thus, under the described conditions, the activity of luciferase was clearly enhanced by PMA treatment for NF-*κ*B and AP-1 activation, and by forskolin exposure for CREB (Figure 3(b)). Interestingly, Cp-BF remarkably diminished the NF-*κ*B-mediated luciferase activity in a dose-dependent manner, whereas others were marginally suppressed (Figure 3(b)). In agreement with this result, treatment with 200 *μ*g/mL Cp-BF decreased the nuclear translocation of p50 but not p65 triggered by LPS treatment in RAW264.7 cells (Figure 3(c)).

### 3.3. Cp-BF Inhibits NF-*κ*B Activation Pathway by Suppressing IKK*β* Activity

**Figure 4:**
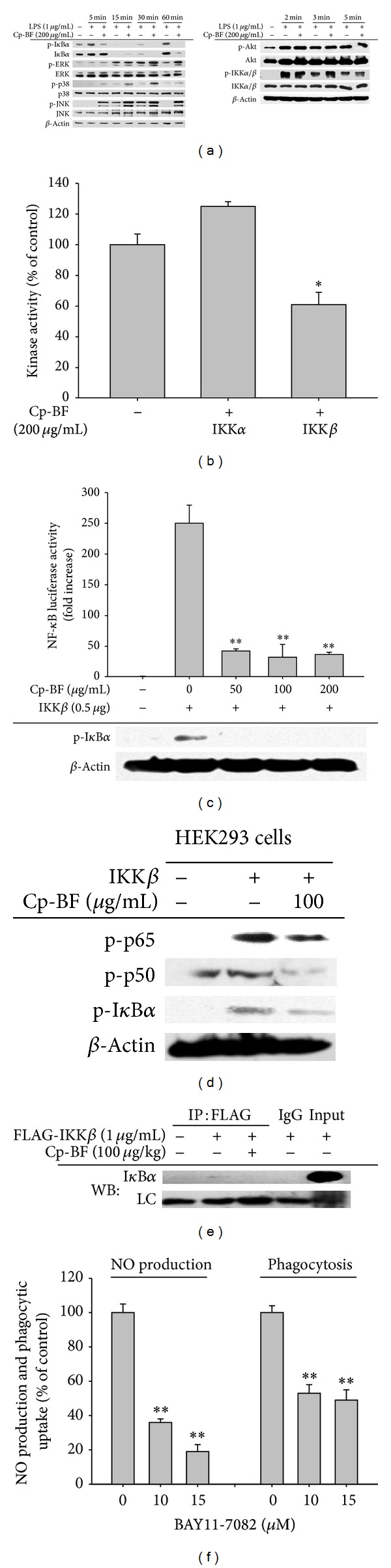
Effect of Cp-BF on the upstream signalling for NF-*κ*B activation. (a) Phosphoprotein or total protein levels of I*κ*B*α*, Akt, p38, ERK, JNK, IKK*α*/*β*, and *β*-actin from cell lysates were determined by phosphospecific or total protein antibodies. (b) Kinase activities of IKK*α* and IKK*β* were determined by a direct kinase assay using purified enzymes. The control was set as 100% for each enzyme activity obtained with vehicle treatment. (c) NF-*κ*B-mediated luciferase activity in IKK*β*-transfected HEK293 cells was measured using a luminometer. Phosphoprotein levels of I*κ*B*α* were determined by immunoblot analysis. (d) Phosphoprotein levels of I*κ*B*α*, p50, and p65 from IKK*β*-transfected RAW264.7 cells were determined by immunoblot analysis. (e) Binding of IKK to I*κ*B*α* was determined by immunoprecipitation and immunoblot analysis of whole cell lysates of LPS-treated RAW264.7 cells (5 × 10^6^ cells/mL). (f) Level of NO was determined by Griess assay with culture supernatants of RAW264.7 cells treated with BAY11-7082 and LPS (1 *μ*g/mL) for 24 h. The effect of BAY11-7082 on phagocytic uptake of RAW264.7 cells treated with FITC-dextran (1 mg/mL) was determined by flow cytometric analysis. *P < 0.05 and **P < 0.01 compared to control.

Immunoblot analysis revealed that Cp-BF was able to suppress the upstream signalling pathway for NF-*κ*B activation. Thus, Cp-BF clearly blocked the phosphorylation of I*κ*B*α* at 5, 30, and 60 min (Figure 4(a) left panel). In contrast, this fraction did not block the phosphorylation of p38, JNK, and ERK from 5 to 60 min (Figure 4(a) left panel). In addition, there was no alteration of phosphorylation levels of Akt and IKK*α*/*β* between 2 to 5 min (Figure 4(a) right panel). Since there was no inhibition of IKK*α*/*β* phosphorylation but suppression of I*κ*B*α* phosphorylation, Cp-BF was assumed to have dampened the enzymatic activities of IKK*α* and IKK*β*. In order to test our theory, we employed kinase assays to evaluate IKK*α*/*β* kinase activity. As shown in Figure 4(b), Cp-BF significantly suppressed the kinase activity of IKK*β*—but not IKK*α*—up to 44% at 200 *μ*g/mL. Similarly, overexpression of IKK*β*-induced luciferase activity and phosphorylation of I*κ*B*α* was completely diminished by Cp-BF (50 to 200 *μ*g/mL) (Figure 4(c)). In agreement with this result, the phosphorylation of NF-*κ*B subunits (p65 and p50) and I*κ*B*α* induced by IKK*β* overexpression in HEK293 cells was strongly suppressed by Cp-BF (Figure 4(d)). Finally, we found that Cp-BF was able to block the molecular interaction between IKK*β* and I*κ*B*α* (Figure 4(e)).

The importance of IKK in inflammatory responses was also demonstrated using the strong IKK inhibitor BAY11-7082. Specifically, BAY11-7082 inhibited the release of NO and suppressed the phagocytic uptake of FITC-dextran (Figure 4(f)).

### 3.4. Cp-BF Ameliorates HCl/EtOH-Induced Gastritis via Suppression of NF-*κ*B Pathway

**Figure 5:**
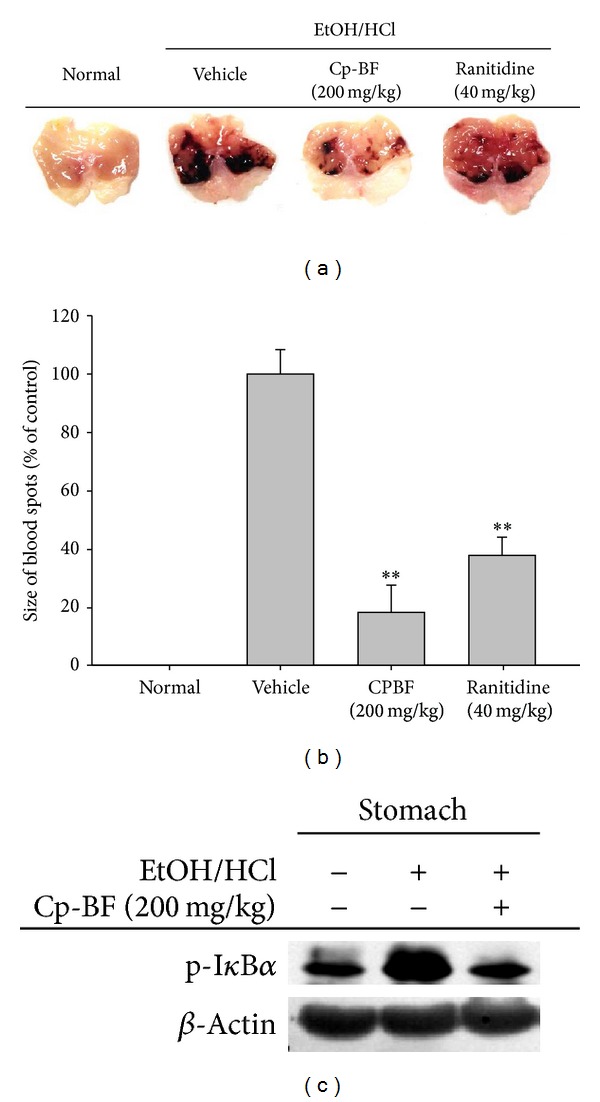
Effect of Cp-BF on inflammatory stomach lesions in HCl/EtOH-treated mice. ((a) and (b)) Mice administered orally with Cp-BF (200 mg/kg) or ranitidine (40 mg/kg) for 3 days were treated with HCl/EtOH delivered orally. After 1 h, gastric lesions in the stomach were photographed and measured with a ruler. Gastric lesions after treatment with inducer alone were set as 100%. (c) Phosphoprotein levels of I*κ*B*α* from tissue lysates were determined using phosphospecific and total protein antibodies; *β*-actin was used as a control. **P < 0.01 compared to control.

To demonstrate the anti-inflammatory activity of Cp-BF* in vivo*, we analysed the effects of orally administered Cp-BF in a model of gastritis. As shown in Figures 5(a) and 5(b), orally administered Cp-BF ameliorated the inflammatory symptoms of* HCl/EtOH-induced gastritis in vivo*. Cp-BF (200 mg/kg) clearly suppressed the formation of inflammatory lesions in stomach during HCl/EtOH treatment and was more effective than ranitidine (40 mg/kg). Interestingly, Cp-BF also suppressed the phosphorylation of I*κ*B*α* induced by gastric damage (Figure 5(c)).

## 4. Discussion

As part of the strategy to develop fruit bodies of artificially cultured* Cordyceps* species,* Cordyceps pruinosa* was selected and cultivation conditions were established with grain rice-containing medium. Recently, our group succeeded in manufacturing fruit bodies of* Cordyceps pruinosa* (Figure 1(a)) at the mass production level, and as the next step we decided to explore its pharmacological activity. Thus, in the present study, we evaluated the anti-inflammatory mechanism of a butanol extract (Cp-BF) of the* Cordyceps pruinosa* fruit body.

Similar to a previous study of a methanol extract of the* Cordyceps pruinosa* fruit body, we found that Cp-BF could suppress the functional activation of macrophages in inflammatory responses. Thus, Cp-BF suppressed the essential outcomes of inflammatory process from macrophage activation such as production of NO and TNF-*α* (Figures 2(a) and 2(b)), ROS generation (Figure 2(c)), and phagocytic uptake (Figure 2(d)), without altering cell viability (Figure 2(e)) [32, 33]. Considering the importance of macrophages in inflammation, these data strongly implied that Cp-BF could negatively modulate macrophage-mediated inflammatory responses. These results are consistent with the previous reports that extracts of* Cordyceps militaris* can diminish inflammatory gene expression in LPS-treated RAW264.7 cells [34]. Furthermore, a chemical derivative of militarin from* Cordyceps militaris* exhibits strong anti-inflammatory properties in LPS-treated macrophages [35]. The ethanol extract of* Cordyceps bassiana* also blocks the production of IL-12 in LPS-treated RAW264.7 cells [36]. Since the majority of inflammatory diseases such as hepatitis, gastritis, colitis, and nephritis are known to be caused by overactivated macrophages, our data strongly suggest that the inhibitory activity of macrophage function may contribute to the immunopharmacological action of* Cordyceps*. In fact, the curative efficacy of these mushrooms against macrophage-mediated diseases has been previously demonstrated in a number of different disease animal models [37, 38].

Understanding the molecular mechanism of Cp-BF mediated suppression of macrophage inflammatory responses is a significant goal for immunopharmacological research of the genus* Cordyceps*. However, only a few reports to date have attempted to explore the molecular target of* Cordyceps* species with respect to their anti-inflammatory actions. Specifically, we previously suggested that the phosphorylation of p38 might target the inhibition of IL-12 expression in* Cordyceps bassiana* [36]. Cordycepin was found to suppress the phosphorylation of Akt for NF-*κ*B inhibition in LPS-treated conditions [39]. It was also reported that NF-*κ*B could be targeted by a methanol extract of* Cordyceps pruinosa*, although no further evaluation of its upstream signalling enzymes was performed [15]. Importantly, none of these reports clearly identified a target protein responsible for the activity of* Cordyceps* as they only tested the phosphorylation levels of relevant pathways. To improve such methodological limitations, we employed a direct kinase assay followed by an overexpression approach for target proteins to confirm their involvement. Through this approach, we identified IKK*β* as a new potential target enzyme of Cp-BF.

We next analysed the mRNA levels of inflammatory genes (Figure 3(a)), luciferase promoter activity (Figure 3(b)), and nuclear levels of transcription factors (Figure 3(c)). Our results suggested that Cp-BF was able to suppress macrophage-mediated inflammatory responses at the transcriptional level by suppression of the NF-*κ*B pathway. Through determination of upstream phosphorylation patterns regulating the activation and translocation of NF-*κ*B, we confirmed the presence of a target event between IKK and I*κ*B*α* based on clear suppression of the phosphorylation of I*κ*B*α* by Cp-BF (Figure 4(a) left panel) and lack of inhibition of upstream phosphorylation events for the activation of IKK and Akt (Figure 4(a) right panel). Although the inhibitory activity was not strong, direct kinase assay with IKK*β* indicated that approximately 40% of IKK*β* enzyme activity was suppressed by Cp-BF (200 *μ*g/mL), implying that IKK*β* may be a potential target enzyme. Indeed, additional experiment data strongly supported the idea. Cp-BF treatment remarkably blocked NF-*κ*B-mediated luciferase activity induced by overexpressed IKK*β* (Figure 4(c)), upregulation of p65/p50 phosphorylation (Figure 4(d)), and molecular binding between IKK and I*κ*B*α* (Figure 4(e)). However, the discrepancy between kinase assay (Figure 4(b)) and IKK overexpression experiments (Figures 4(c) and 4(d)) should be further explored. Presently, we speculate that there might be different pharmacological sensitivities between purified human IKK*β* used for kinase assays and transfected IKK*β* expressed in HEK293 cells.

The ethnopharmacological value of* Cordyceps* is now greatly accepted in Asian countries, since these mushrooms have been traditionally prescribed for the treatment of various inflammatory and metabolic diseases. Unlike ginseng, one of most famous herbal plants worldwide, for which cultivation methods have been traditionally established, the majority of* Cordyceps* products are still harvested in nature. Although natural fruit bodies of* Cordyceps* may be best in terms of pharmacological activities, large-scale production remains a key issue. Therefore, recent studies with* Cordyceps* species have focused on the development of cultural methods for mass production of* Cordyceps*. In fact, our group has established a suitable cultivation method and plant manufacturing system that achieves high productivity of* Cordyceps* fruit bodies [40]. Using the approach, we analysed the pharmacological efficacies of artificially cultivated fruit bodies of* Cordyceps militaris*,* Cordyceps bassiana*, and* Cordyceps pruinosa* maintained under identical conditions.

To specifically test the activity of* Cordyceps pruinosa*, we employed a mouse* in vivo* disease model with HCl/EtOH-induced gastritis under oral administration of Cp-BF. Interestingly, this fraction strongly ameliorated gastritis symptoms (Figures 5(a) and 5(b)) and suppressed the phosphorylation of I*κ*B*α* increased by HCl/EtOH treatment (Figure 5(c)). These results strongly implied that the artificially cultivated fruit bodies exhibit similar pharmacological activity as the natural forms of this mushroom. Indeed, we previously observed that the artificially cultivated fruit body of* Cordyceps bassiana* can effectively treat symptoms of atopic dermatitis [41]. Moreover, the artificially cultivated fruit body of* Cordyceps militaris* possesses antihepatitis activity (data not shown). Taken together, these results strongly suggest that the artificially cultivated fruit bodies of* Cordyceps pruinosa* can be developed for the purpose of pharmaceutical and nutraceutical remedies.

To date little information on the phytochemical characteristics of* Cordyceps pruinosa* has been reported. A total of 5 alcohols, 21 amino acids, 15 organic acids, 4 purines, 3 pyrimidines, 7 sugars, 11 fatty acids, and 5 other metabolites in* Cordyceps pruinosa* mycelia cultivated with various media have been characterized by metabolic profile analysis [42]. This same study also showed that* Cordyceps pruinosa* mycelia contains a high phenolic content and exhibits good antioxidative properties, although the specific compounds responsible for this effect have not yet been identified [42]. According to the analysis of cordycepin by HPLC, this compound might not be included in this mushroom (Figure 1(c)). Therefore, future studies will need to include the understanding of active components with anti-inflammatory activity in this mushroom.

**Figure 6:**
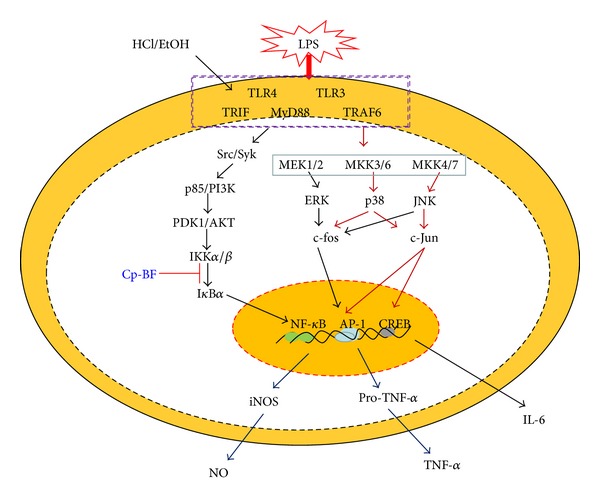
Putative inhibitory pathway of the Cp-BF-mediated anti-inflammatory response.

In summary, we have shown that Cp-BF can block macrophage-mediated inflammatory responses such as NO and TNF-*α* production, ROS generation, and phagocytic uptake at the transcriptional level. Orally administered Cp-BF clearly ameliorates gastric ulcer formation induced by HCl/EtOH treatment. By analysing transcription factors and intracellular signalling enzymes, our results suggest that Cp-BF can directly inhibit IKK linked to the suppression of NF-*κ*B pathway as summarized in Figure 6. The data from the present study strongly support the pharmaceutical and nutraceutical values of artificially cultivated fruit bodies of* Cordyceps pruinosa*.

## Figures and Tables

**Table 1:** PCR primers used in this study.

Name		Sequence (5′ to 3′)
iNOS	F	CCCTTCCGAAGTTTCTGGCAGCAG
	R	GGCTGTCAGAGCCTCGTGGCTTTGG
TNF-*α*	F	TTGACCTCAGCGCTGAGTTG
	R	CCTGTAGCCCACGTCGTAGC
IL-6	F	GTACTCCAGAAGACCAGAGG
	R	TGCTGGTGACAACCACGGCC
GAPDH	F	CACTCACGGCAAATTCAACGGCA
	R	GACTCCACGACATACTCAGCAC

## Abbreviations

Cp-BF: Butanol fraction of* Cordyceps pruinosa*
NO: Nitric oxide
iNOS: Inducible NO synthase
TNF-*α*: necrosis factor-alpha
ERK: Extracellular signal-related kinase
TLR: Toll-like receptor
MAPK: Mitogen activated protein kinase
NF-*κ*B: Nuclear factor-*κ*B
AP-1: Activator protein-1
JNK: c-Jun N-terminal kinase
Akt: Protein kinase B
IKK: I*κ*B*α* kinase
ELISA: Enzyme-linked immunosorbent assay
MTT: 3-(4,5-dimethylthiazol-2-yl)-2,5-diphenyltetrazolium bromide
PI3K: Phosphoinositide 3-kinases
LPS: Lipopolysaccharide
RT-PCR: Reverse transcriptase-polymerase chain reaction.
